# Design, Synthesis and Biological Evaluation of Novel Bromophenol Derivatives Incorporating Indolin-2-One Moiety as Potential Anticancer Agents

**DOI:** 10.3390/md13020806

**Published:** 2015-02-02

**Authors:** Li-Jun Wang, Shuai-Yu Wang, Bo Jiang, Ning Wu, Xiang-Qian Li, Bao-Cheng Wang, Jiao Luo, Meng Yang, Shui-Hua Jin, Da-Yong Shi

**Affiliations:** Institute of Oceanology, Chinese Academy of Sciences, Qingdao 266071, China; E-Mails: wanglijun@qdio.ac.cn (L.-J.W.); 12-12sy@163.com (S.-Y.W.); jiangbo@qdio.ac.cn (B.J.); wuning@qdio.ac.cn (N.W.); lnu101@163.com (X.-Q.L.); 13365456626@163.com (B.-C.W.); luojiao2012@163.com (J.L.); qwyangmeng@163.com (M.Y.); shisheng14226@126.com (S.-H.J.)

**Keywords:** bromophenol, indolin-2-one, anticancer, molecular hybrid, structure–activity relationship

## Abstract

A series of bromophenol derivatives containing indolin-2-one moiety were designed and evaluated that for their anticancer activities against A549, Bel7402, HepG2, HeLa and HCT116 cancer cell lines using MTT assay* in vitro*. Among them, seven compounds (**4g**–**4i**, **5h**, **6d**, **7a**, **7b**) showed potent activity against the tested five human cancer cell lines. Wound-healing assay demonstrated that compound **4g** can be used as a potent compound for inactivating invasion and metastasis by inhibiting the migration of cancer cells. The structure–activity relationships (SARs) of bromophenol derivatives had been discussed, which were useful for exploring and developing bromophenol derivatives as novel anticancer drugs.

## 1. Introduction

Cancer is a significant health problem throughout the world, which is a leading cause of death. It is estimated that 14.1 million new cancer cases and 8.2 million cancer-related deaths occurred in 2012 according to the WHO report. The incidence of cancer has been increasing in most regions of the world, which will further increase to 19.3 million new cancer cases per year by 2025 [1]. Although many effective anticancer agents have been developed, therapies for cancer are less than satisfactory due to side effects. Therefore, novel effective agents for treating cancer disease are urgently needed today.

**Figure 1:**
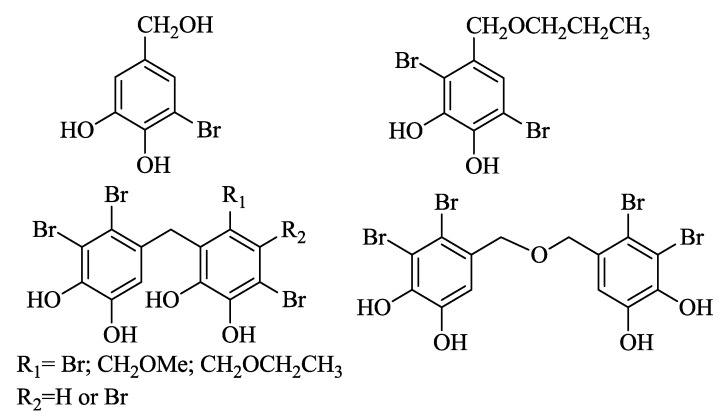
Structures of bromophenols possessing potent activity against cancer.

Bromophenols, natural marine products isolated from various marine biology, are known to possess various potent activities including antioxidative, protein tyrosine kinase (PTK) inhibitory, anticancer, protein tyrosine phosphatase 1B inhibitory, antithrombotic, antimicrobial, anti-inflammatory [2], which have attracted much attention, also due to their unique structures. For example, 3-bromo-4,5-dihydroxybenzyl alcohol (Figure 1) showed significant cytotoxicity to KB cells (IC_50_ = 8.9 μg/mL) [3]. 2,5-dibromo-3,4-dihydroxybenzyl n-propyl ether (Figure 1) synthesized from natural bromophenol demonstrated potent activity against DLD-1 and HCT-116 Cells lines with IC_50_ values 1.72 and 0.80 µM, respectively [4]. Xu and co-workers reported a series of natural bromophenols (3,4-dibromo-5(methoxymethyl)-1,2-benzenediol, Figure 1) which showed vigorous activities against KB, Bel7402, HELF and A549 cancer cells with IC_50_ values under 10 μg/mL [5]. In our previous study, a variety of bromophenols (Figure 1) isolated from various marine algae, which exhibited excellent anticancer activity against A549, BGC-823, MCF-7, B16-BL6, HT-1080, A2780, Bel7402 and HCT-8 human cancer cell lines, could be used as potent antitumor agents for PTK over-expression of c-kit and is considered as part of a new therapeutic strategy for treatment of cancer [6].

**Figure 2:**
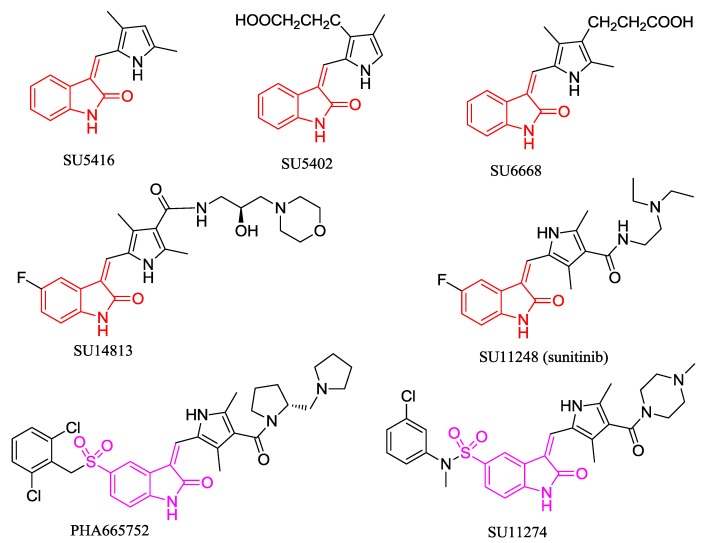
Selected indolinone derivatives as anticancer agents in preclinical or clinical trials.

The structural scaffold of oxindole is proven as a core structure in various active compounds that exhibit a wide variety of biological activities including anti-inflammatory [7,8], tuberculosis [9], antiglycation [10], antitumor [11,12,13,14], neurological disorders [15], antioxidant [16], antimicrobial [17], and anticonvulsant activity [18]. Among them, indolin-2-one derivatives have aroused great attention for their anticancer activities and were developed as antitcancer drugs, such as SU5416, SU5402, SU6668and SU14813 (Figure 2), which have been reported as potent and selective inhibitors of different protein kinases and showed a significant cytotoxic activity [19,20]. SU5416 is the first selective kinase insert domain receptor (KDR) inhibitor used in clinical trials. Its derivative SU11248 (sunitinib, Figure 2), an inhibitor of RTK approved by the US Food and Drug Administration (FDA), has been approved and marketed for the treatment of gastrointestinal stromal cancers and renal cell carcinoma [21]. SU11274 and PHA665752 (Figure 2) containing 3-methylene indolin-2-one scaffold with sulfonyl group located on the 5-position were proved as an effective inhibitor of Met tyrosine kinase and have entered preclinical and multi-center clinical studies for anticancer activity [22,23]. Therefore, the indolin-2-one moiety is the pharmacophore in developing anticancer agents.

Molecular hybrids with two pharmacophores often lead to synergistic activity. It has been observed that the incorporation of indolin-2-one moiety into the active anticancer moiety (such as pyrrole, oxadiazole, thiazolidinone,* etc*.) demonstrated profound growth inhibitory activity against different cancer cells, which was an efficient strategy for thedesign of novel antitumor agents [24,25,26].

In view of these observations, a novel series of bromophenols derivatives containing indolin-2-one moiety was designed and evaluated for* in vitro* anticancer activity against cancer cell lines (Human lung cancer cell line, A549; Human hepatoma cell lines, Bel-7402 and HepG2; human cervical cancer cell line, HeLa; human colon cancer cell line, HCT116) using MTT assay. The most potent activity compound **4g** on cancer cell migration was investigated by using the wound-healing assay. Structure–activity relationships (SARs) of these bromophenols analogs are also discussed in this paper.

## 2. Results and Discussion

### 2.1. Chemistry

**Scheme 1:**
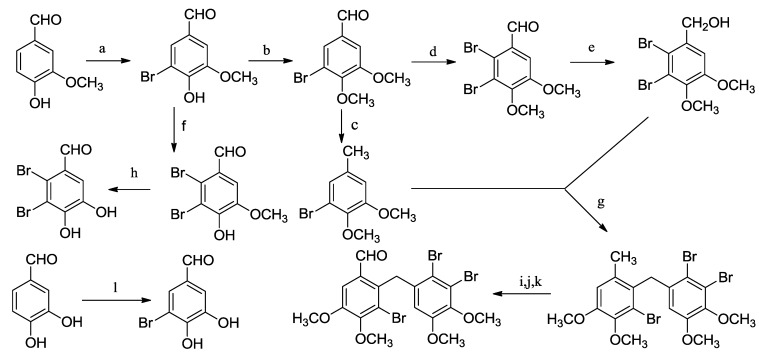
Synthesis of aldehydes. Reagents and conditions: (**a**) Br_2_, CH_3_OH, 0 °C; (**b**) CH_3_I, K_2_CO_3_, DMF, room temperature (rt); (**c**) 80% N_2_H_4_·H_2_O, KOH, diglycol, 120 °C; (**d**) Br_2_, AcOH, 60 °C; (**e**) NaBH_4_, CH_3_OH, 0 °C; (**f**) Br_2_, AcOH, Fe, reflux, 12 h; (**g**) AlCl_3_, CH_2_Cl_2_, rt; (**h**) BBr_3_, DCM, −78 °C; (**i**) NBS, AIBN, CCl_4_, hv; (**j**) K_2_CO_3_, 1,4-dioxane, H_2_O, 90 °C; (**k**) PCC, DCM, rt; (**l**) Br_2_, AcOH, rt.

**Scheme 2:**
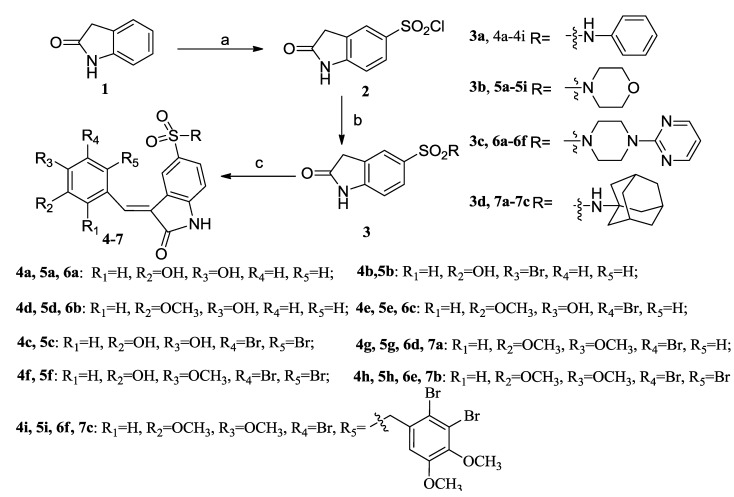
Synthesis of bromophenol derivatives **4**–**7**. Reagents and conditions: (**a**) ClSO_3_H, 65 °C, 1 h; (**b**) R_1_R_2_NH, THF, reflux; (**c**) EtOH, aldehyde, piperidine, reflux.

The synthetic procedures for the preparation of aldehydes (Scheme 1) have been publised in our previous reports [27,28,29,30]. As shown in Scheme 2, a series of bromophenol derivatives were synthesized by Knoevenagel condensation between different substituted indolin-2-one and the corresponding aldehydes in order to explore the SARs of these derivatives and obtain the potencial lead compounds. Firstly, oxindole (**1**) was reacted with ClSO_3_H to yield compound **2**. Then, compound **2** and amines were heated for 3 h in tetrahydrofuran (THF) at 80 °C to afford 5-substituted-indolin-2-one (**3**). At last, the reaction between 5-substituted-indolin-2-one (**3**) and the synthesized aldehydes was performed under the condition of Knoevenagel condensation in ethanol with a catalytic amount of piperidine to give the desired bromophenol derivatives **4**–**7** in good yields. All of the synthesized derivatives were purified and their structures were characterized by spectroscopic means (^1^H, ^13^C NMR, MS and HRMS).

### 2.2. Anti-Cancer Activity

#### 2.2.1. Antiproliferative Activity

**Figure 3:**
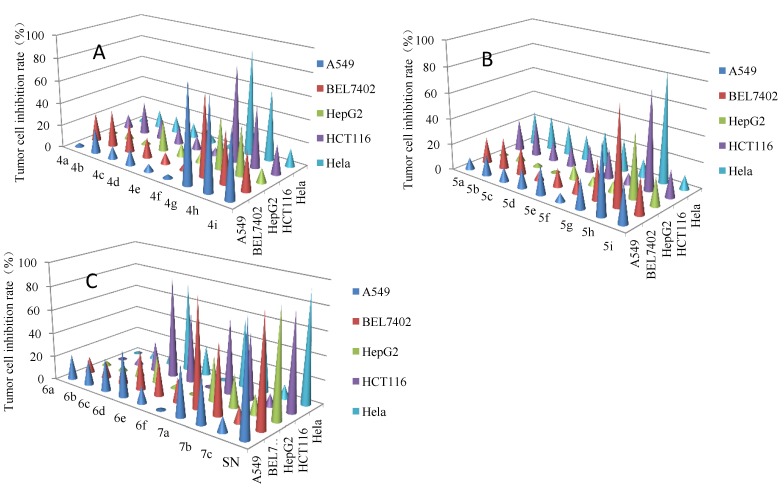
Inhibitory activity of bromophenol derivatives (**A**: compounds **4a**–**4i**; **B**: compounds **5a**–**5i**; **C**: compounds **6a**–**6f**, **7a**–**7c** and Sunitinib) against five human cancer cell lines; the percent inhibition rate of tumor cell at 10 µg/mL inhibitor; the cells were seeded in a 96-well plate and incubated overnight. Then, the cells were treated with various amounts of compounds and incubated for 48 h. Cell proliferation was evaluated with the MTT Assay. Sunitinib (SN) as the positive control.

All the synthesized compounds were investigated for their anticancer activity* in vitro* on five human cancer cell lines, namely against A549, Bel7402, HepG2, HeLa and HCT116 cancer cell lines using MTT assay and sunitinib as a positive control. The results of inhibited ration of comounds **4**–**7** were listed in Figure 3, and the IC_50_ values of selective compounds (**4g**–**4i**, **5h**, **6d**, **6e**, **7a**, **7b**) were listed in Table 1.

As shown in Figure 3, compounds **4g**–**4i**, **5h**, **6d**, **6e**, **7a** and **7b** exhibited potent anticancer activity against A549, Bel7402, HepG2, HeLa and HCT116 cancer cell lines at 10 μg/mL, respectively. Of them, compound **4g** showed significant activity against A549, Bel7402, HepG2, HeLa and HCT116 cancer cell lines with the inhibition ratio of 88.2%, 71.9%, 85.2% and 93.0% at 10 μg/mL, respectively, which were comparable to those of sunitinib. Compounds **4a**–**4c**, **5a**–**5c**, and **6a** with two hydroxy groups on phenol ring showed weaker activity against of five cancer cells at 10 μg/mL. After methylation of 3-hydroxyl group on phenol ring of compounds **4d**–**4f**, **5d**–**5f**, **6b**, and **6c**, the activity have not obviously changed (**4d** *vs.* **4a**, **4e** *vs.* **4b**, **4f** *vs.* **4c**, **5d** *vs.* **5a**, **5e** *vs.* **5b**, **5f** *vs.* **5c**, **6b**, **6c** *vs.* **6a**).

**Table 1:** IC_50_ values of selected bromophenol derivatives against five human cancer cell lines.

Compd	IC_50_ (μg/mL) *^a^*				
	A549	Bel7402	HepG2	Hct116	Hela
**4g**	6.6 ± 0.82	9.2 ± 0.84	13.2 ± 2.42	9.1 ± 0.13	7.4 ± 0.22
**4h**	14.4 ± 1.86	12.3 ± 0.23	14.3 ± 0.86	9.8 ± 0.55	8.3 ± 0.67
**4i**	9.9 ± 0.11	22.3 ± 1.11	NA *^b^*	NA	NA
**5h**	10.1 ± 0.72	9.7 ± 2.35	11.2 ± 1.26	8.6 ± 0.26	18.0 ± 0.13
**6d**	25.4 ± 0.82	18.6 ± 0.91	NA	5.6 ± 0.42	17.6 ± 0.89
**6e**	NA	NA	NA	9.8 ± 0.20	NA
**7a**	12.5 ± 0.19	7.9 ± 0.26	25.0 ± 0.18	6.1 ± 0.23	8.6 ± 0.14
**7b**	12.5 ± 0.45	12.5 ± 0.39	14.2 ± 0.77	8.2 ± 0.54	9.3 ± 0.47
TTEDM *^c^*	NA	44.1 ± 1.19	NA	32.3 ± 2.03	23.5 ± 1.41
Sunitinib *^d^*	10.4± 0.54	3.2 ± 0.15	4.5 ± 0.23	2.8 ± 0.27	1.6 ± 0.08

*^a^* IC_50_: Concentration of the compound producing 50% cell growth inhibition after 48 h of drug exposure, as determined by the MTT assay. Each experiment was run at least three times, and the results are presented as average values ± standard deviation; *^b^* NA: Compound showing IC_50_ value > 50 μg/mL; *^c^* 2,2',3-tribromo-3',4,4',5-tetrahydroxy-6'-ethyloxymethyldiphenylmethane (TTEDM) is a marine bromophenol compound derived from marine *algae. P*.; *^d^* Sunitinib as the positive control.

It was inspired that methylation of 4-hydroxyl group on phenol ring (**4g**, **4h**, **5g**, **5h**, **6d**, **6e**, **7a**, **7b**) could significantly enhance their anti-cancer activity, which indicated that the hydrophobic parameter may affect their anticancer activity. However, compounds **4i**, **5i**, **6f**, **7c** with four methoxy groups in diphenyl methane moiety showed low anticancer activity, suggesting that many of the hydrophobic groups have a deleterious effect. The steric hindrance may affect their activity also. The derivatives of mono-brominated on phenol ring exhibited better activity than those of di-brominated compounds (**4b** *vs.* **4c**, **4e** *vs.* **4f**, **4g** *vs.* **4h**, **5b** *vs.* **5c**, **5e** *vs.* **5f**, **6d** *vs.* **6e**, **7a** *vs.* **7b**). On the contrary, compound **5h** with di-brominated on phenol moiety showed better inhibitory activity against cancer cells than those of compound **5g** with mono-brominated on phenol moiety. These results indicated that the number of bromine atom on phenol moiety could affect the anticancer activities of these hybrid derivatives. Furthermore, it is notable that most compounds introducing secondary amino groups in 5-position of indolin-2-one (**4a**–**4i** and **7a**–**7c**) displayed significant activity compared to those of compounds (**5a**–**6f**) with tertiary sulfonamide groups, indicating that types of amino groups at 5-position of indolin-2-one could influence the activities of these derivatives. From Table 1, it is also seenthat these synthesized bromophenol derivatives containing indolin-2-one moiety (**4g**–**4i**, **5h**, **6d**, **6e**, **7a**, **7b**) showed better activities than 2,2',3-tribromo-3',4,4',5-tetrahydroxy-6'-ethyloxymethyldiphenylmethane (TTEDM) which was a marine bromophenol compound isolated from marine algae* Polysiphonia urcedata.*

#### 2.2.2. Inhibitory Effect of Compound **4g** on Cancer Cell Migration

**Figure 4:**
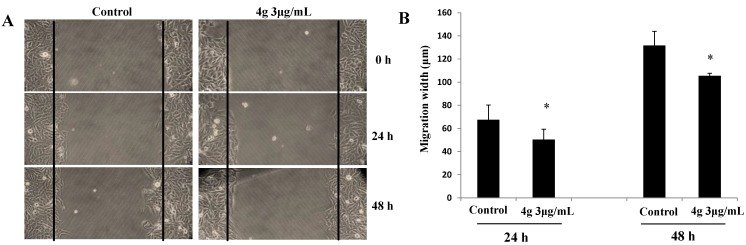
(**A**) The HepG2 cells were scraped by a pipette tip to generate wounds. After treatment with or without **4g** 3 μg/mL in the presence of 1 μg/mL Mitomycin C, cells migration was recorded by microscopy at 0, 24, 48 h; (**B**) Results were obtained from three separate experiments. The bar represents the mean ± SE, * (*p* < 0.05) indicates a significant difference between the control and **4g** treated samples.

Cancer cell invasion, a hallmark of cancer, plays a critical role during the carcinomas arising from epithelial tissues progressed to higher pathological grades of malignancy [31]. To further investigate the anticancer effect of **4g**, we studied the inhibitory effect on cancer cell migration; an invasive process often observed in cancer by using the wound-healing assay [32]. We found that the migration of HepG2 cells was significantly inhibited by 48 h treatment with **4g** at 3 μg/mL concentration compared to the untreated control (Figure 4A).

The migration width (the distances of migrated cells) of cancer cells treated with/without **4g** was summarized in Figure 4B. It was demonstrated that ~20% of cell migration was inhibited by treatment with **4g** at 3 μg/mL concentration for 48 h. These results suggested that **4g** might be used as a potent compound for inactivating invasion and metastasis by inhibiting the migration of cancer cells.

## 3. Experimental Section

### 3.1. Chemistry

^1^H and ^13^C NMR spectra were recorded on Bruker DRX 500 MHz spectrometers with tetramethylsilane (TMS) as the internal standard (Bruker, Bremerhaven, Germany). MS and HRMS spectra were determined on a LCMS-IT-TOF mass spectrometer (Shimadzu, Kyoto, Japan). Column chromatography (CC): Silica gel (200–300 mesh; Qingdao Makall Group CO., Ltd; Qingdao; China). All reactions were monitored using thin-layer chromatography (TLC) on silica gel plates. Reaction reagents were purchased from J&K Scientific Ltd. (Chaoyang District, Beijing, China) Organic solvents were analytical reagent grade and purchased from Tianjin Chemical Reagent Co., Ltd. (Jinnan District, Tianjin, China). The synthesized compounds were named using ChemBioDraw Ultra software (version 12.0, Waltham, MA, USA).

#### General Procedure for the Preparation of Derivatives (**4**–**7**)

To a 100 mL flask charged with chlorosulfonic acid (25 mL), 2-oxindole (50 mmoL) at 0 °C was added slowly. After the addition, the reaction mixture was stirred at room temperature for 1.5 h. Then, the reaction mixture was heated to 68 °C for 1 h, cooled, and poured into ice water (200 mL). The precipitate was washed with water and dried in a vacuum oven to give 5-chlorosulfonyl-2-oxindole (**2**) which was used without further purification.

A mixture of 5-chlorosulfonyl-2-oxindole (5 mmoL) and appropriate amine (10 mmoL) in tetrahydrofuran (THF, 50 mL) was heated to refluxing and stirred for 3 h. Then, this mixture was concentrated under reduced pressure, and HCl (pH = 3, 25 mL) was added and stirred for 15 min. The crude product was filtered, washed with ice water (100 mL) and dried in a vacuum oven to give 5-sulfamoyl-2-oxindole (**3**) which was used without further purification.

Piperidine (50 μL) was added to a mixture of compound **3** (0.5 mmoL) and appropriate aldehyde (0.55 mmoL) in ethanol (5 mL). The reaction mixture was heated to refluxing and stirred for 2 h, and TLC analysis indicated when the reaction was complete. The crude product was filtered, washed with ethanol and dried in a vacuum (if no solid precipitated, the crude product was chromatographed using a silica gel column) to afford the title compound **4**–**7** as a yellow solid.

(*E*)-*N*-(4-Bromophenyl)-3-(3,4-dihydroxybenzylidene)-2-oxoindoline-5-sulfonamide (**4a**). Yield: 66.1%; ^1^H NMR (DMSO-*d*_6_, 500 MHz, ppm): δ 10.96 (s, 1H), 10.21 (s, 1H), 8.10 (s, 1H), 7.75 (s, 1H), 7.58 (dd, 1H, *J* = 1.5, 8.5 Hz), 7.56 (s, 1H), 7.38–7.40 (overlap, 3H), 7.06 (d, 1H, *J* = 9.0 Hz), 7.00 (d, 1H, *J* = 9.0 Hz), 6.97 (d, 2H, *J* = 8.5 Hz); ^13^C NMR (DMSO-*d*_6_, 125 MHz, ppm): δ 169.4, 150.3, 148.9, 146.1, 145.2, 137.7, 140.2, 132.4 (2C), 128.8, 125.4, 123.2, 122.5, 122.3 (2C), 121.9, 120.8, 118.0, 116.5, 116.3, 110.5; ESIMS: *m*/*z* 484 [M − H]^−^ HRESIMS: Calc for C_21_H_15_BrN_2_O_5_S [M − H]^−^ 484.9805, found 484.9812.

(*E*)-3-(3-Bromo-4,5-dihydroxybenzylidene)-*N*-(4-bromophenyl)-2-oxoindoline-5-sulfonamide (**4b**). Yield: 67.6%; ^1^H NMR (DMSO-*d*_6_, 500 MHz, ppm): δ 11.00 (s, 1H), 10.24(s, 1H), 8.11 (s, 1H), 7.79(s, 1H), 7.56(dd, 1H, *J* = 1.5, 8.5 Hz), 7.54 (s, 1H), 7.39 (d, 2H, *J* = 9.0 Hz), 7.32 (s, 1H), 7.06 (d, 2H, *J* = 9.0 Hz), 6.99 (d, 1H, *J* = 8.5 Hz); ^13^C NMR (DMSO-*d*_6_, 125 MHz, ppm): δ 169.1, 147.4, 146.6, 145.8, 139.7, 137.9, 132.4 (2C), 132.1, 131.7,126.6, 126.0, 124.8, 122.7, 122.0 (2C), 121.6, 116.3, 116.1, 110.7, 110.4; ESIMS: *m*/*z* 562 [M − H]^−^ HRESIMS: Calc for C_21_H_13_BrN_2_O_5_S [M − H]^−^ 562.8915, found 562.8917.

(*E*)-*N*-(4-Bromophenyl)-3-(2,3-dibromo-4,5-dihydroxybenzylidene)-2-oxoindoline-5-sulfonamide (**4c**). Yield: 77.4%; ^1^H NMR (DMSO-*d*_6_, 500 MHz, ppm): δ 11.08 (s, 1H), 10.30 (s, 1H), 7.74 (s, 1H), 7.55 (d, 1H, *J* = 8.5 Hz), 7.54 (s, 1H), 7.33 (d, 2H, *J* = 8.5 Hz), 7.15(s, 1H), 6.96 (d, 1H, *J* = 8.5 Hz), 6.93 (d, 2H, *J* = 8.5 Hz); ^13^C NMR (DMSO-*d*_6_, 125 MHz, ppm): δ 168.7, 147.0, 146.9, 146.0, 138.0, 137.6, 132.4 (2C), 132.1, 129.6, 126.7, 126.0, 121.8 (2C), 121.5, 121.2, 116.4, 115.8, 115.3, 114.4, 110.8; ESIMS: *m*/*z* 640 [M − H]^−^ HRESIMS: Calc for C_21_H_12_Br_3_N_2_O_5_S [M − H]^−^ 640.8014, found 640.8022.

(*E*)-*N*-(4-Bromophenyl)-3-(4-hydroxy-3-methoxybenzylidene)-2-oxoindoline-5-sulfonamide (**4d**). Yield: 68.0%; ^1^H NMR (DMSO-*d*_6_, 500 MHz, ppm): δ 11.12 (s, 1H), 10.30 (s, 1H), 8.18 (s, 1H), 7.60 (s, 1H), 7.56 (dd, 1H, *J* = 1.5, 8.5 Hz), 7.32 (s, 1H), 7.31 (d, 1H, *J* = 8.5 Hz), 7.24 (d, 2H, *J* = 8.5 Hz), 6.99 (d, 1H, *J* = 8.5 Hz), 6.92 (d, 1H, *J* = 8.5 Hz), 6.87 (d, 2H, *J* = 8.5 Hz), 3.83 (s, 3H); ^13^C NMR (DMSO-*d*_6_, 125 MHz, ppm): δ 169.6, 150.8, 148.2, 147.8, 145.5, 140.8, 139.6, 132.0 (2C), 128.5, 125.4, 124.8, 122.9, 122.5 (2C), 122.2, 120.7, 116.4, 116.1, 113.4, 110.1, 55.9; ESIMS: *m*/*z* 498 [M − H]^−^ HRESIMS: Calc for C_22_H_16_BrN_2_O_5_S [M − H]^−^ 498.9970, found 498.9969.

(*E*)-3-(3-Bromo-4-hydroxy-5-methoxybenzylidene)-*N*-(4-bromophenyl)-2-oxoindoline-5-sulfonamide (**4e**). Yield: 73.5%; ^1^H NMR (DMSO-*d*_6_, 500 MHz, ppm): δ 11.08 (s, 1H), 10.30 (s, 1H), 7.92 (s, 1H), 7.55(s, 1H), 7.48 (s, 1H), 7.47 (s, 1H), 7.37 (d, 2H, *J* = 8.5 Hz), 7.32 (d, 1H, *J* = 8.5 Hz), 7.05 (d, 2H, *J* = 8.5 Hz), 6.97 (d, 1H, *J* = 8.5 Hz); ^13^C NMR (DMSO-*d*_6_, 125 MHz, ppm): δ 168.0, 150.3, 144.8, 141.7, 140.8, 138.3, 138.2, 132.3 (2C), 131.2, 128.0, 124.2, 123.1, 121.9 (2C), 119.7, 116.0, 115.9, 113.7, 111.0, 108.7, 55.8; ESIMS: *m*/*z* 576 [M − H]^−^ HRESIMS: Calc for C_22_H_15_Br_2_N_2_O_5_S [M − H]^−^ 576.9062, found 576.9074.

(*E*)-*N*-(4-Bromophenyl)-3-(2,3-dibromo-4-hydroxy-5-methoxybenzylidene)-2-oxoindoline-5-sulfonamide (**4f**). Yield: 68.9%; ^1^H NMR (DMSO-*d*_6_, 500 MHz, ppm): δ 11.08 (s, 1H), 10.24 (s, 1H), 7.68 (s, 1H), 7.58 (d, 1H, *J* = 8.5 Hz), 7.55 (s, 1H), 7.53 (s, 1H),7.32 (d, 2H, *J* = 8.5 Hz), 6.97 (d, 1H, *J* = 8.5 Hz), 6.92 (d, 2H, *J* = 8.5 Hz), 3.87 (s, 3H); ^13^C NMR (DMSO-*d*_6_, 125 MHz, ppm): δ 168.5, 152.6, 147.1, 146.0, 137.7, 137.6, 132.4 (2C), 132.2, 131.4, 129.8, 126.8, 122.1 (2C), 121.6, 121.2, 116.9, 116.4, 115.6, 114.2, 110.9, 65.4; ESIMS: *m*/*z* 654 [M − H]^−^ HRESIMS: Calc for C_22_H_14_Br_3_N_2_O_5_S [M − H]^−^ 654.8168, found 654.8179.

(*E*)-3-(3-Bromo-4,5-dimethoxybenzylidene-*N*-(4-bromophenyl)-2-oxoindoline-5-sulfonamide (**4g**). Yield: 67.2%; ^1^H NMR (DMSO-*d*_6_, 500 MHz, ppm): δ 11.08 (s, 1H), 10.24 (s, 1H), 7.63 (s, 1H), 7.58–7.61 (overlap, 2H), 7.50 (s, 1H), 7.40 (s, 1H), 7.31 (d, 2H, *J* = 8.5 Hz), 7.05 (d, 1H, *J* = 9.0 Hz), 6.94 (d, 2H, *J* = 8.5 Hz), 3.87 (s, 3H), 3.84 (s, 3H); ^13^C NMR (DMSO-*d*_6_, 125 MHz, ppm): δ 168.9, 153.8, 148.3, 147.5, 137.3, 132.3 (2C), 132.1, 131.5, 131.4, 129.6, 126.7, 126.3, 122.4 (2C), 121.4, 121.3, 117.6, 116.5, 113.5, 110.7, 60.8, 56.6; ESIMS: *m*/*z* 590 [M − H]^−^ HRESIMS: Calc for C_23_H_17_Br_2_N_2_O_5_S [M − H]^−^ 590.9219, found 590.9230.

(*E*)-*N*-(4-Bromophenyl)-3-(2,3-dibromo-4,5-dimethoxybenzylidene)-2-oxoindoline-5-sulfonamide (**4h**). Yield: 82.5%; ^1^H NMR (DMSO-*d*_6_, 500 MHz, ppm): δ 11.16 (s, 1H), 10.24 (s, 1H), 7.66 (s, 1H), 7.60 (d, 1H, *J* = 8.5 Hz), 7.57 (s, 1H), 7.52 (s, 1H), 7.31 (d, 2H, *J* = 8.5 Hz), 6.99 (d, 1H, *J =* 8.5 Hz), 6.90 (d, 2H, *J =* 8.5 Hz), 3.86 (s, 3H), 3.82 (s, 3H); ^13^C NMR (DMSO-*d*_6_, 125 MHz, ppm): δ 168.5, 152.8, 148.6, 147.2, 137.5, 136.8, 132.4 (2C), 132.2, 131.7, 130.0, 128.0, 122.2 (2C), 122.1, 121.7, 121.2, 117.0, 116.6, 113.9, 110.9, 60.9, 60.7; ESIMS: *m*/*z* 668 [M − H]^−^ HRESIMS: Calc for C_23_H_16_Br_3_N_2_O_5_S [M − H]^−^ 668.8334, found 668.8335.

(*E*)-3-(3-Bromo-2-(2,3-dibromo-4,5-dimethoxybenzyl-4,5-dimethoxybenzylidene)-*N*-(4-bromophenyl)-2-oxoindoline-5-sulfonamide (**4i**). Yield: 88.2%; ^1^H NMR (DMSO-*d*_6_, 500 MHz, ppm): δ 10.99 (s, 1H), 10.35 (s, 1H), 7.63 (s, 2H), 7.52 (s, 1H), 7.32-7.34 (overlap, 3H), 6.92-6.95 (overlap, 3H), 6.33 (s, 1H), 4.22 (s, 2H), 3.85(s, 3H), 3.80(s, 3H), 3.58(s, 3H), 3.31(s, 3H); ^13^C NMR (DMSO-*d*_6_, 125 MHz, ppm): δ 168.3, 152.4, 152.3, 147.5, 146.9, 145.8, 137.6, 136.9, 136.2, 132.3 (3C), 131.2, 130.5, 129.8, 127.8, 122.5, 121.6 (3C), 121.5, 121.3, 117.0, 116.2, 113.2, 112.9, 110.7, 60.8, 60.2, 56.7, 56.1, 41.3; ESIMS: *m*/*z* 896 [M − H]^−^ HRESIMS: Calc for C_32_H_25_Br_4_N_2_O_7_S [M − H]^−^ 896.8107, found 896.8121.

(*E*)-3-(3,4-Dihydroxybenzylidene)-5-(morpholinosulfonyl)indolin-2-one (**5a**). Yield: 52.3%; ^1^H NMR (DMSO-*d*_6_, 500 MHz, ppm): δ 11.04 (s, 1H), 8.04 (s, 1H), 7.63 (s, 1H), 7.56 (dd, 1H, *J* = 1.5, 8.5 Hz), 7.14 (s, 1H), 7.06–7.11 (overlap, 2H), 6.87 (d, 1H, *J* = 8.5 Hz), 3.62 (t, 4H, *J* = 4.5 Hz), 2.84 (t, 4H, *J* = 4.5 Hz); ^13^C NMR (DMSO-*d*_6_, 125 MHz, ppm): δ 169.4, 148.9, 145.9, 145.1, 140.2, 129.8, 126.8, 125.4, 123.2, 122.3, 121.5, 121.1, 117.5, 116.2, 110.6, 65.7 (2C), 46.5 (2C); ESIMS: *m*/*z* 401 [M − H]^−^ HRESIMS: Calc for C_19_H_17_N_2_O_6_S [M − H]^−^ 401.0813, found 401.0813.

(*E*)-3-(3-Bromo-4,5-dihydroxybenzylidene)-5-(morpholinosulfonyl)indolin-2-one (**5b**). Yield: 66.3%; ^1^H NMR (DMSO-*d*_6_, 500 MHz, ppm): δ 11.10 (s, 1H), 8.08 (s, 1H), 7.60 (s, 1H), 7.54 (dd, 1H, *J* = 1.5, 8.0 Hz), 7.42 (s, 1H), 7.07 (s, 1H), 7.01 (d, 1H, *J* = 8.0 Hz), 3.62 (t, 4H, *J* = 4.5 Hz), 2.84(t, 4H, *J* = 4.5 Hz); ^13^C NMR (DMSO-*d*_6_, 125 MHz, ppm): δ 169.1, 147.0, 145.8, 144.3, 140.0, 130.0, 129.4, 126.8, 126.3, 124.7, 122.7, 119.4, 116.5, 110.7, 110.3, 65.7 (2C), 46.5 (2C); ESIMS: *m*/*z* 478 [M − H]^−^ HRESIMS: Calc for C_19_H_16_BrN_2_O_6_S [M − H]^−^ 478.9901, found 478.9918.

(*E*)-3-(2,3-Dibromo-4,5-dihydroxybenzylidene)-5-(morpholinosulfonyl)indolin-2-one (**5c**). Yield: 68.3%; ^1^H NMR (DMSO-*d*_6_, 500 MHz, ppm): δ 10.95 (s, 1H), 7.79 (s, 1H), 7.67 (s, 1H), 7.55 (dd, 1H, *J* = 1.5, 8.5 Hz), 7.10 (s, 1H), 6.95 (d, 1H, *J* = 8.5 Hz), 3.66 (t, 4H, *J* = 4.5 Hz), 2.85 (t, 4H, *J* = 4.5 Hz); ^13^C NMR (DMSO-*d*_6_, 125 MHz, ppm): δ 169.7, 146.2, 146.0, 144.4, 139.7, 129.9 128.7, 127.3, 126.6, 125.8, 122.2, 121.9, 117.3, 114.8, 110.2, 65.9 (2C), 46.0 (2C*)*; ESIMS: *m*/*z* 556 [M − H]^−^ HRESIMS: Calc for C_19_H_15_Br_2_N_2_O_6_S [M − H]^−^ 556.9015, found 556.9023.

(*E*)-3-(4-Hydroxy-3-methoxybenzylidene)-5-(morpholinosulfonyl)indolin-2-one (**5d**). Yield: 77.5%; ^1^H NMR (DMSO-*d*_6_, 500 MHz, ppm): δ 11.03 (s, 1H), 8.00(s, 1H), 7.69 (s, 1H), 7.52(dd, 1H, *J* = 1.5, 8.5 Hz), 7.38 (s, 1H), 7.26 (dd, 1H, *J* = 1.5, 8.0 Hz), 7.09 (d, 1H, *J* = 8.0 Hz), 6.90 (d, 1H, *J* = 8.0 Hz), 3.84 (s, 3H), 3.62 (t, 4H, *J* = 4.5 Hz), 2.86(t, 4H, *J* = 4.5 Hz); ^13^C NMR (DMSO-*d*_6_, 125 MHz, ppm): δ 169.8, 150.4, 147.8, 144.3, 140.4, 129.9, 127.2, 125.7, 125.6, 123.6, 122.5, 121.6, 116.7, 113.9, 110.0, 66.0 (2C), 56.4, 46.7 (2C); ESIMS: *m*/*z* 415 [M − H]^−^ HRESIMS: Calc for C_20_H_19_N_2_O_6_S [M − H]^−^ 415.0955, found 415.0969.

(*E*)-3-(3-Bromo-4-hydroxy-5-methoxybenzylidene)-5-(morpholinosulfonyl)indolin-2-one (**5e**). Yield: 67.2%; ^1^H NMR (DMSO-*d*_6_, 500 MHz, ppm): δ 11.10 (s, 1H), 8.07 (s, 1H), 7.67 (s, 1H), 7.59 (s, 1H), 7.55 (d, 1H, *J* = 8.0 Hz), 7.40 (s, 1H), 7.04 (d, 1H, *J* = 8.0 Hz), 3.90 (s, 3H), 3.62 (t, 4H, *J* = 4.5 Hz), 2.86 (t, 4H, *J* = 4.5 Hz); ^13^C NMR (DMSO-*d*_6_, 125 MHz, ppm): δ 169.1, 148.7, 148.0, 144.2, 139.8, 131.0, 128.8, 127.0, 126.4, 124.9, 123.0, 119.3, 115.6, 109.8, 109.3, 65.7 (2C), 56.6, 46.4 (2C); ESIMS: *m*/*z* 493 [M − H]^−^ HRESIMS: Calc for C_20_H_18_BrN_2_O_6_S [M − H]^−^ 493.0073, found 493.0074.

(*E*)-3-(2,3-Dibromo-4-hydroxy-5-methoxybenzylidene)-5-(morpholinosulfonyl)indolin-2-one (**5f**). Yield: 78.3%; ^1^H NMR (DMSO-*d*_6_, 500 MHz, ppm): δ 10.95 (s, 1H), 8.02 (s, 1H), 7.67 (s, 1H), 7.56 (dd, 1H, *J* = 1.5, 8.0 Hz), 7.39 (s, 1H), 7.04 (d, 1H, *J* = 8.0 Hz), 3.90 (s, 3H), 3.62 (t, 4H, *J* = 4.5 Hz), 2.86 (t, 4H, *J* = 4.5 Hz); ^13^C NMR (DMSO-*d*_6_, 125 MHz, ppm): δ 169.2, 148.8, 147.5, 144.3, 139.8, 130.9 127.1, 126.4, 124.9, 123.0, 121.9, 121.7, 119.3, 113.1, 109.9, 65.7 (2C), 56.6, 46.4 (2C); ESIMS: *m*/*z* 570 [M − H]^−^ HRESIMS: Calc for C_20_H_17_Br_2_N_2_O_6_S [M − H]^−^ 570.9172, found 570.9180.

(*E*)-3-(3-Bromo-4,5-dimethoxybenzylidene)-5-(morpholinosulfonyl)indolin-2-one (**5g**). Yield: 82.6%; ^1^H NMR (DMSO-*d*_6_, 500 MHz, ppm): δ 11.00 (s, 1H), 8.07 (s, 1H), 7.70 (s, 1H), 7.62 (dd, 1H, *J =* 1.5, 8.5 Hz), 7.60 (s, 1H), 7.46 (s, 1H), 7.05 (d, 1H, *J* = 8.5 Hz), 3.89 (s, 3H), 3.82 (s, 3H), 3.62 (t, 4H, *J* = 4.5 Hz), 2.87 (t, 4H, *J* = 4.5 Hz); ^13^C NMR (DMSO-*d*_6_, 125 MHz, ppm): δ 168.9, 153.9, 148.3, 144.8, 138.7, 131.7, 130.7, 126.9, 126.0, 125.5, 122.1, 121.7, 116.6, 114.4, 110.1, 65.7 (2C), 60.8, 56.8, 46.4 (2C); ESIMS: *m*/*z* 507 [M − H]^−^ HRESIMS: Calc for C_21_H_20_BrN_2_O_6_S [M − H]^−^ 507.0227, found 507.0231.

(*E*)-3-(2,3-Dibromo-4,5-dimethoxybenzylidene)-5-(morpholinosulfonyl)indolin-2-one (**5h**). Yield: 88.2%; ^1^H NMR (CDCl_3_, 500 MHz, ppm): δ 10.96 (s, 1H), 7.89 (s, 1H), 7.67 (dd, 1H, *J* = 1.5, 8.5 Hz), 7.63 (s, 1H), 7.29 (s, 1H), 7.11 (d, 1H, *J* = 8.5 Hz) , 3.97 (s, 3H) , 3.93 (s, 3H), 3.75 (t, 4H, *J* = 4.5 Hz), 2.93 (t, 4H, *J* = 4.5 Hz); ^13^C NMR (CDCl_3_, 125 MHz, ppm): δ 169.7, 153.0, 149.4, 147.1, 138.6, 131.9, 130.8, 128.3, 128.0, 123.0, 122.9, 122.0, 117.9, 112.9, 111.0, 66.3 (2C), 61.1, 56.8, 46.3 (2C); ESIMS: *m*/*z* 584 [M − H]^−^ HRESIMS: Calc for C_21_H_19_Br_2_N_2_O_6_S [M − H]^−^ 584.9325, found 584.9336.

(*E*)-3-(3-Bromo-2-(2,3-dibromo-4,5-dimethoxybenzyl)-4,5-dimethoxybenzylidene)-5-(morpholinosulfonyl) indolin-2-one (**5i**). Yield: 84.3%; ^1^H NMR (DMSO-*d*_6_, 500 MHz, ppm): δ 10.96 (s, 1H), 7.62 (s, 1H), 7.53 (dd, 1H, *J* = 1.5, 8.5 Hz), 7.31 (s, 1H), 7.21 (s, 1H), 6.99 (d, 1H, *J* = 8.5 Hz), 6.56 (s, 1H), 4.26 (s, 2H), 3.80 (s, 3H), 3.79 (s, 3H), 3.60 (t, 4H, *J* = 4.5 Hz), 3.54 (s, 3H), 3.52 (s, 3H), 2.75 (t, 4H, *J* = 4.5 Hz); ^13^C NMR (DMSO-*d*_6_, 125 MHz, ppm): δ 168.2, 152.3 (2C), 147.4, 147.1, 145.8, 137.5, 136.8, 131.5, 130.7, 130.6, 127.9, 126.9, 122.6, 122.3, 121.6, 121.3, 116.7, 113.7, 113.4, 110.6, 65.7 (2C), 60.7, 60.1, 56.9, 56.2, 41.4, 46.2 (2C); ESIMS: *m*/*z* 812 [M − H]^−^ HRESIMS: Calc for C_30_H_28_Br_3_N_2_O_8_S [M − H]^−^ 812.9113, found 812.9122.

(*E*)-3-(3,4-Dihydroxybenzylidene)-5-((4-(pyrimidin-2-yl)piperazin-1-yl) sulfonyl) indolin-2-one (**6a**). Yield: 55.1%; ^1^H NMR (DMSO-*d*_6_, 500 MHz, ppm): δ 11.11 (s, 1H), 8.30 (d, 2H, *J* = 5.0 Hz), 7.77 (s, 1H), 7.57 (s, 1H), 7.52 (d, 1H, *J* = 8.0 Hz), 7.14 (s, 1H), 7.02 (d, 1H, *J* = 8.0 Hz), 6.94 (d, 1H, *J* = 8.0 Hz), 6.73 (d, 1H, *J* = 8.0 Hz),6.60 (t, 1H, *J* = 5.0 Hz), 2.95 (t, 4H, *J* = 4.5 Hz), 2.92 (t, 4H, *J* = 4.5 Hz); ^13^C NMR (DMSO-*d*_6_, 125 MHz, ppm): δ 167.9, 161.2, 158.4 (2C), 147.1, 145.9, 143.0, 141.1, 130.5, 127.7, 126.5, 125.4, 122.9, 117.6, 116.4, 115.8, 111.1, 110.2, 109.0, 46.2 (2C), 43.0 (2C); ESIMS: *m*/*z* 478 [M − H]^−^ HRESIMS: Calc for C_23_H_20_N_5_O_5_S [M − H]^−^ 478.1184, found 478.1191.

(*E*)-3-(4-Hydroxy-3-methoxybenzylidene)-5-((4-(pyrimidin-2-yl) piperazin-1-yl) sulfonyl) indolin-2-one (**6b**). Yield: 61.5%; ^1^H NMR (DMSO-*d*_6_, 500 MHz, ppm): δ 11.00 (s, 1H), 8.30 (d, 2H, *J* = 5.0 Hz), 8.00 (s, 1H), 7.68 (s, 1H), 7.58 (d, 1H, *J* = 8.5 Hz), 7.39 (s, 1H), 7.05 (d, 1H, *J* = 8.5 Hz), 6.99 (d, 1H, *J* = 8.0 Hz), 6.87 (d, 1H, *J* = 8.0 Hz), 6.60 (t, 1H, *J* = 5.0 Hz), 3.84 (s, 3H), 2.94 (t, 4H, *J* = 4.5 Hz), 2.89 (t, 4H, *J* = 4.5 Hz); ^13^C NMR (DMSO-*d*_6_, 125 MHz, ppm): δ 167.9, 161.2, 158.4 (2C), 150.1, 147.5, 144.0, 141.4, 129.8, 127.2, 126.3, 125.3, 123.4, 121.5, 121.3, 116.7, 115.7, 111.1, 110.7, 56.1, 46.2 (2C), 43.0 (2C); ESIMS: *m*/*z* 492 [M − H]^−^ HRESIMS: Calc for C_24_H_22_N_5_O_5_S [M − H]^−^ 492.1341, found 492.1347.

(*E*)-3-(3-Bromo-4-hydroxy-5-methoxybenzylidene)-5-((4-(pyrimidin-2-yl)piperazin-1-yl) sulfonyl) indolin-2-one (**6c**). Yield: 70.2%; ^1^H NMR (DMSO-*d*_6_, 500 MHz, ppm): δ 10.70 (s, 1H), 8.29 (d, 2H, *J* = 5.0 Hz), 7.88 (s, 1H), 7.70 (s, 1H), 7.50 (s, 1H),7.36 (d, 1H, *J* = 8.0 Hz), 7.14 (s, 1H), 6.91 (d, 1H, *J* = 8.0 Hz), 6.58 (t, 1H, *J* = 5.0 Hz), 3.81 (s, 3H), 2.97 (t, 4H, *J* = 4.5 Hz), 2.91 (t, 4H, *J* = 4.5 Hz); ^13^C NMR (DMSO-*d*_6_, 125 MHz, ppm): δ 168.0, 161.2, 158.4 (2C), 150.4, 145.1, 142.0, 141.4, 128.4, 126.2, 125.1, 123.5, 120.0, 117.8, 116.7, 113.7, 111.0, 1109.8, 108.7, 55.7, 46.2 (2C), 43.0 (2C); ESIMS: *m*/*z* 570 [M − H]^−^ HRESIMS: Calc for C_24_H_21_BrN_5_O_5_S [M − H]^−^ 570.0433, found 570.0452.

(*E*)-3-(3-Bromo-4,5-dimethoxybenzylidene)-5-((4-(pyrimidin-2-yl)piperazin-1-yl) sulfonyl) indolin-2-one (**6d**). Yield: 76.2%; ^1^H NMR (DMSO-*d*_6_, 500 MHz, ppm): δ 11.11 (s, 1H), 8.31 (d, 2H, *J* = 5.0 Hz), 8.05 (s, 1H), 7.68(s, 1H), 7.58-7.60 (overlap, 2H), 7.45 (s, 1H), 7.01 (d, 1H, *J* = 8.5 Hz), 6.60 (t, 1H, *J* = 5.0 Hz), 3.89(s, 3H), 3.82(s, 3H), 2.95 (t, 4H, *J* = 4.5 Hz), 2.91 (t, 4H, *J* = 4.5 Hz); ^13^C NMR (DMSO-*d*_6_, 125 MHz, ppm): δ 168.9, 161.2, 158.4 (2C), 154.0, 148.3, 144.8, 137.3, 131.7, 130.7, 127.6, 127.3, 125.5, 121.7, 122.0, 116.6, 114.4, 111.1, 110.1, 60.8, 56.8, 46.1 (2C), 42.9 (2C); ESIMS: *m*/*z* 584 [M − H]^−^ HRESIMS: Calc for C_25_H_23_BrN_5_O_5_S [M − H]^−^ 584.0606, found 584.0609.

(*E*)-3-(2,3-Dibromo-4,5-dimethoxybenzylidene)-5-((4-(pyrimidin-2-yl)piperazin-1-yl) sulfonyl) indolin-2-one (**6e**). Yield: 78.3%; ^1^H NMR (Pyridine-*d*_5_, 500 MHz, ppm): δ 10.16 (s, 1H), 8.14 (d, 2H, *J* = 5.0 Hz), 7.99 (s, 1H), 7.67 (d, 1H, *J* = 8.0 Hz), 7.42 (s, 1H), 7.03 (s, 1H), 6.97 (d, 1H, *J* = 8.0 Hz), 6.25 (t, 1H, *J* = 5.0 Hz), 3.84 (s, 3H), 3.78 (s, 3H), 3.04 (t, 4H, *J* = 4.5 Hz), 2.90 (t, 4H, *J* = 4.5 Hz); ^13^C NMR (Pyridine-*d*_5_, 125 MHz, ppm): δ 169.6, 161.6, 158.2 (2C), 153.4, 150.3, 148.3, 137.3, 132.4, 131.0, 128.9, 128.4, 123.2, 123.1, 122.3, 118.2, 113.8, 110.93, 110.86, 60.9, 57.0, 46.5 (2C), 43.2 (2C); ESIMS: *m*/*z* 661 [M − H]^−^ HRESIMS: Calc for C_25_H_22_Br_2_N_5_O_5_S [M − H]^−^ 661.9690, found 661.9714.

(*E*)-3-(3-Bromo-2-(2,3-dibromo-4,5-dimethoxybenzyl)-4,5-dimethoxybenzylidene)-5-((4-(pyrimidin-2-yl) piperazin-1-yl) sulfonyl) indolin-2-one (**6f**). Yield: 85.2%; ^1^H NMR (DMSO-*d*_6_, 500 MHz, ppm): δ 10.90 (s, 1H), 8.31 (d, 2H, *J* = 5.0 Hz), 7.59 (s, 1H), 7.53 (dd, 1H, *J* = 1.5, 8.5 Hz), 7.28 (s, 1H), 7.21 (s, 1H), 6.96 (d, 1H, *J* = 8.5 Hz), 6.60–6.62 (overlap, 2H), 4.26 (s, 2H), 3.83 (s, 3H), 3.80 (s, 3H), 3.54 (s, 3H), 3.51 (s, 3H), 2.92 (t, 4H, *J* = 4.5 Hz), 2.81 (t, 4H, *J* = 4.5 Hz); ^13^C NMR (DMSO-*d*_6_, 125 MHz, ppm): δ 168.2, 161.1, 158.4 (2C), 152.3, 152.2, 147.3, 147.0, 145.7, 137.6, 137.0, 131.5, 130.6, 127.7, 127.2, 122.7, 122.2, 121.6, 121.2, 116.6, 114.0, 113.4, 111.1, 110.5, 60.7, 60.0, 56.9, 56.2, 46.1 (2C), 42.8 (2C), 41.5; ESIMS: *m*/*z* 889 [M − H]^−^ HRESIMS: Calc for C_34_H_31_Br_3_N_5_O_7_S [M − H]^−^ 889.9522, found 889.9500.

(*E*)-*N*-(Adamantan-1-yl)-3-(3-bromo-4,5-dimethoxybenzylidene)-2-oxoindoline-5-sulfonamide (**7a**). Yield: 42.6%; ^1^H NMR (DMSO-*d*_6_, 500 MHz, ppm): δ 11.09 (s, 1H), 8.07 (s, 1H), 7.78–7.71 (overlap, 2H), 7.66 (s, 1H), 7.46 (s, 1H), 7.02 (d, 1H, *J* = 8.0 Hz), 3.94 (s, 3H), 3.93 (s, 3H), 1.99 (m, 6H), 1.57 (m, 3H), 1.25 (m, 6H); ^13^C NMR (DMSO-*d*_6_, 125 MHz, ppm): δ 168.1, 153.8, 148.3, 144.7, 138.2, 137.6, 130.9, 130.1, 126.7, 126.0, 121.7, 121.6, 118.0, 115.3, 110.3, 60.8, 56.3, 55.3, 43.1 (3C), 35.8 (3C), 29.5 (3C); ESIMS: *m*/*z* 571 [M − H]^−^ HRESIMS: Calc for C_27_H_28_BrN_2_O_5_S [M − H]^−^ 571.0907, found 571.0908.

(*E*)-*N*-(Adamantan-1-yl)-3-(2,3-dibromo-4,5-dimethoxybenzylidene)-2-oxoindoline-5-sulfonamide (**7b**). Yield: 52.5%; ^1^H NMR (DMSO-*d*_6_, 500 MHz, ppm): δ 11.08 (s, 1H), 7.85 (s, 1H), 7.70 (d, 1H, 8.0 Hz), 7.64 (s, 1H), 7.28 (s, 1H), 7.01(d, 1H, *J* = 8.0 Hz), 3.80 (s, 3H) , 3.83 (s, 3H), 1.85 (m, 6H), 1.70 (m, 3H), 1.40 (m, 6H); ^13^C NMR (DMSO-*d*_6_, 125 MHz, ppm): δ 168.6, 152.9, 148.4, 146.1, 136.3, 132.1, 129.6, 128.8, 128.6, 121.9, 121.4, 120.8, 116.6, 113.9, 111.7, 60.8, 56.9, 54.1, 42.9 (3C), 35.9 (3C), 29.3 (3C); ESIMS: *m*/*z* 649 [M − H]^−^ HRESIMS: Calc for C_27_H_27_Br_2_N_2_O_5_S [M − H]^−^ 648.9996, found 649.0013.

(*E*)-*N*-(Adamantan-1-yl)-3-(3-bromo-2-(2,3-dibromo-4,5-dimethoxybenzyl)-4,5-dimethoxybenzylidene)-2-oxoindoline-5-sulfonamide (**7c**). Yield: 57.6%; ^1^H NMR (CDCl_3_, 500 MHz, ppm): δ 10.00 (s, 1H), 9.43 (s, 1H), 7.87 (s, 1H), 7.69 (s, 1H), 7.61 (d, 1H, *J* = 8.0 Hz), 7.26 (s, 1H), 7.21 (s, 1H), 7.16 (d, 1H, *J* = 8.0 Hz), 4.29 (s, 2H), 3.90 (s, 3H), 3.75 (s, 3H), 3.54 (s, 3H), 3.52 (s, 3H), 2.03 (m, 6H), 1.75 (m, 3H), 1.25 (m, 6H); ^13^C NMR (CDCl_3_, 125 MHz, ppm): δ 169.4, 152.5, 152.3, 148.4, 146.1, 144.7, 137.1, 135.4, 131.4, 129.2 130.6, 127.7, 127.6, 123.0, 122.3, 121.6, 121.3, 117.6, 111.8, 111.6, 110.1, 60.9, 60.4, 56.5, 56.1, 55.3, 46.9, 43.1 (3C), 35.8 (3C), 29.5 (3C); ESIMS: *m*/*z* 876 [M − H]^−^ HRESIMS: Calc for C_36_H_36_Br_3_N_2_O_7_S [M − H]^−^ 876.9793, found 876.9793.

### 3.2. Antiproliferative Activities Assays

#### 3.2.1. Cell Culture and Proliferation Assay

The cells including Human lung cancer cell line, A549; Human hepatoma cell lines, Bel-7402 and HepG2; human cervical cancer cell line, HeLa; human colon cancer cell line, HCT116 were purchased from BOSTER, Ltd (Wuhan, China). The cells were maintained in DMEM supplemented with 10% FBS and 1% antibiotics at 37 °C and in an atmosphere containing 5% CO_2_. The cells were split 1:3 when they reached 80%–90% confluence. Cell proliferation was measured using the 3-(4,5-dimethylthiazol-2-yl)-2,5-diphenyltetrazolium bromide (MTT) colorimetric assay. The IC_50_ was calculated by using the SPSS 17.0 (SPSS, Inc., Chicago, IL, USA).

The cells (4 × 10^3^ cells/well) with 10% FBS culture medium were seeded in a 96-well plate and incubated overnight. Next, the cells were treated with various amounts of compounds and incubated for 48 h. Subsequently, 20 μL of 5 mg/mL MTT was transferred into each well, and the cells were incubated for 4 h. The medium in each well was carefully removed, and 150 μL DMSO was then added to each well. The samples were thoroughly agitated for 10 min on a shaker. Finally, the absorbance of the samples at 490 and 690 nm was measured against a background control (blank) using a microplate reader.

#### 3.2.2. Wound-Healing Assay

The HepG2 cells (1 × 10^5^ cells/well) were cultured in 6-well plates rinsed with PBS and then starved overnight in 2% FBS medium until they reached 95% confluence. A single wound was then scratched in the center of the cell monolayers with a 200 μL sterile plastic pipette tip. The wounded monolayers were washed twice to remove the non-adherent cells and were incubated with various concentrations of compound **4g** in the presence of 1 μg/mL of mitomycin C. To measure the length of the endothelial cells that had migrated from the edge of the injured monolayer, images were obtained immediately after wounding and after a 24, 48 h incubation period, using a phase-contrast microscope (Olympus, Tokyo, Japan). The length was measured by the Image-Pro Plus v 6.0 (Media Cybernetics, Inc., Bethesda, MD, USA). Each experiment was repeated at least three times.

#### 3.2.3. Statistical Analysis

All the experiments were performed at least three times, and the data are presented as mean ± SD values. Differences between the mean values were assessed using one-way analysis of variance. For all the analyses, *p* < 0.05 was considered significant. Statistical analyses were performed using SPSS 17.0 (SPSS, Inc., Chicago, IL, USA).

## 4. Conclusions

**Figure 5:**
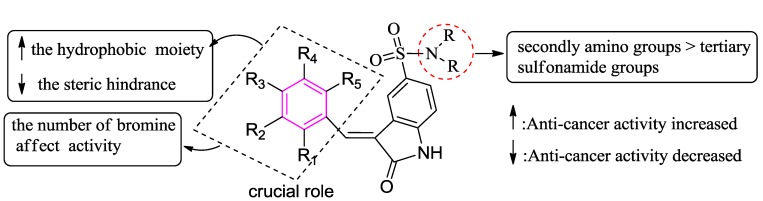
Structure–Activity relationships of bromophenol derivatives anticancer activity.

In summary, a series of bromophenols derivatives containing indolin-2-one moiety were designed and evaluated for their anticancer activities against A549, Bel7402, HepG2, HeLa and HCT116 cancer cell lines using MTT assay* in vitro*. The preliminary SAR analysis (summarized in Figure 5) reveals that: (i) the hydrophobic parameter may affect their anticancer activity; (ii) the steric hindrance may also affect their activity; (iii) the number of bromine atoms on phenol moiety could affect the anticancer activities of these hybrid derivatives; (iv) the types of amino groups at 5-position of indolin-2-one could influence the activities; (v) the bromophenol moiety played a crucial role in maintaining anticancer activities of the conjugated derivatives. Among them, seven compounds (**4g**–**4i**, **5h**, **6d**, **7a**, **7b**) showed potent activity. Wound-healing assay demonstrated that compound **4g** can be used as a potent compound for inactivating invasion and metastasis by inhibiting the migration of cancer cells. These active derivatives targeting properties needs to be further investigated. Potentially, this finding may aid in the design of novel agents for the intervention of cancer.
